# Assessment Hepatomegaly and liver Enzymes in 100 Patients with beta Thalassemia Major in Mashhad, Iran

**Published:** 2012-09-22

**Authors:** H Hashemizadeh, R Noori, SH kolagari

**Affiliations:** 1 Department of nursing, Quchan Branch, Islamic Azad University, Quchan, Iran.; 2 Department of midwifery, Quchan Branch, Islamic Azad University, Quchan, Iran.; 3 Faculty Member of Nursing & Midwifery College, University of Medical sciencesand Health Services, Golestan, Iran.

**Keywords:** Epidemiology, Hepatomegaly, Liver, beta-Thalassemia

## Abstract

**Background:**

Frequent blood transfusion in patients with beta thalassemia major can lead to iron overload especially in liver. Chronic iron overload could cause cirrhosis of the liver. Transfusion- transmitted hepatitis B and C also could develop cirrhosis in individuals.

**Materials and Methods:**

The present cross- sectional descriptive study is to assess hepatomegaly and liver enzymes in 100 patients with beta thalassemia major, ages between 2-18 years old. The study was carried out retrospectively. One hundred medical records have chosen from 400 samples of thalassemia major patients, who are under a regular care of the department of sarvar clinic.

**Results:**

Out of these patients, 55% were male and 45% female. The mean age of thalassemia patients was 10.8± 4.4 years. The mean and S. D of hemoglobin, ferritin, deferoxamine dosage was 8.5 ± 1.5g/dl , 2183 ± 1528 ng , 30 ± 11.16 mg/kg, respectively. Forty six percent of them had hepatomegaly. The mean and S. D of AST and ALT were 95± 70 IU/L and 70 ±35U/L respectively. Splenectomy was performed on 44% of patient.

**Conclusion:**

Hepatomegaly is one of the most common findings in the thalassemic patient that induced with hemosiderosis and hepatitis.

## Introduction

Thalassemia is a problem of 60 countries with the highest prevalence in the Mediterranean region, parts of North and West Africa, the Middle East, the Indian subcontinent, southern Far East and southeastern Asia (1, 2). Iran has a large number of thalassemic patients (3), which about 19,000 cases suffer from major and/or minor thalassemia in Iran (4).

Beta-thalassemia represents a group of recessively inherited hemoglobin disorders first described by Cooley and Lee (5). The disease is characterized by reduced synthesis of β-globin chain. The homozygous state results in severe anemia, which needs a regular blood transfusion (6, 7). On the other hand, frequent blood transfusion can lead to iron overload, which may result in hypogonadism, diabetes mellitus, hypothyroidism, hypoparathyroidism and other endocrine abnormalities (8). In recent years, several authors reported that a high incidence of abnormalities in children, adolescents and young adults suffering from thalassemia major (9, 10). 

Transfusion-associated viral hepatitis resulting in cirrhosis or portal hypertension also may be occurred (11, 12). 

In Italy 50% of thalassemic patients were estimated to have died before the age of 12 years (13). Cornell Medical Center reported a median survival of 17.1 years in patients from 1960 to 1976(14).The advent of safe transfusions has drastically prolonged the life of these patients. However, repeated transfusions have various complications and iron overload (15).

Abdominal examination may reveal changes in the liver, gallbladder, and spleen. Hepatomegaly related to significant extramedullary hematopoiesis typically is observed. Patients who have received blood transfusions may have hepatomegaly or chronic hepatitis due to iron overload (16). 

Frequent blood transfusion can lead to iron overload especially in liver. Liver has a large capacity to produce proteins, which bind the iron and store it in the form of ferritine and haemosiderin, therefore, it can produce sever iron overload. Chronic iron overload may lead to cirrhosis. Transfusion- transmitted hepatitis B and C also can develop to cirrhosis. Second leading cause of death in this patient after 15 years of old is cirrhosis. In these times most of them affected with cirrhosis, hepatitis B and C (17)*.*

This study was aimed to assess the frequency of the hepatomegaly and liver enzymes in 100 patients with major beta thalassemia age between 2 – 18 years old, who attended to Sarvar clinic of Mashhad University of Medical Science**. **

## Method and Materials

This work is a descriptive cross- sectional study. The study was carried out retrospectively. Medical records of all patients aged between 2- 18 years were used from 400 records of major beta thalassemia patients in Sarvar clinic, which were 100 cases. Frequency of the hepatomegaly, liver enzymes (ALT and AST), Hb, ferritin, deferoxamine dosage, age of splenectomy, and age of starting deferoxamine were extracted from the files. The normal range of AST, ALT was considered less than 35 IU/Lit. All patients had received blood transfusions since infancy. 

Liver enzymes levels were measured by commercial kits on the Hitachi 704 analyzer (Hitachi, Tokyo, Japan). 

**Statistical analysis**

The data was recorded in a questionnaire and analyzed using SPSS v.16. 

## Results

The patients were 55 (55%) male and 45 (45%) female. The mean age of thalassemic patients and age of starting the blood transfusion was 9±6 and 10/8± 4/4 months respectively. The mean and S. D of hemoglobin, ferritine, deferoxamine dosage was 8.5 ± 1.5g /dl , 2183 ± 1528 ng (269-6450) , 30 ± 11.16 mg /kg .

Only 33 (33%) of patients maintained their hemoglobin levels >9.0 g/dl. and 37 (37%) Patients had ferritin levels >2,000 ng/ml. Forty six (46%) of them have hepatomegaly. Splenectomy was performed on 44(44%) of patient. 

The mean and S. D of AST and ALT were 95 +/- 70 IU/L, 70 +/- 35U/L respectively. Sixty children (60%) used daily acid folic, and 12(12 %) vit C. Analyzed data revealed that there is no significant difference between the mean of hemoglobin, ferritin, deferoxamine dosage, AST, ALT and hepatomegaly among men and women. There was a significant relation between age of starting transfusion and age of starting deferoxamine (r=0.31 p =0.002). There was a significant relation between level of hemoglobin and deferoxamine dosage (r=0.3 p =0.005).

Hepatomegaly in patients with ages more than 10 years was reported in 34 cases (74%), but in less than 10 years was found in 12 cases (26%) (P-value=0.003).There is no relation between hepatomegaly and the level of ferritin, Hb, gender and splenectomy.There is a significant relation between hepatomegaly with splenomegaly (P-value=0.018), and also with usage of folic acid (0.046).

## Discussion

The mean and S. D of hemoglobin was 8.5 ± 1.5g / dl. Yin and et al reported 44.6% of patients maintained their hemoglobin levels >9.0 g/dl (18). In this research only 33 % of patients maintained their hemoglobin levels >9.0 g/dl. Shamshirzan et al reported hemoglobin level before transfusion about 9.6±2.3 g/dl (19). Researchers in Germany and Bangkok reported hemoglobin level before transfusion about 8.5 and 7.7±1.1 respectively (20, 21). The mean and S. D of hemoglobin in these researches are similar to other studies (20, 21). 

The mean and S. D of ferritine were 2183 ± 1528 (269-6450) ng. Yin reported the mean serum ferritin level 3,143 ng/ml (18). Khawla reported the mean serum ferritin level 2597.2 ± 1976.8 μg/l (22). Li reported serum ferritin levels from the minimum of 1500 ng/mL up to a maximum of 11491 ng/mL (23). Belhoul reported the mean serum ferritin level 2597.2 ± 1976.8 μg/l (24). 

Cunningham reported ferritin levels from 147 to 11 010 ng/mL (median, 1696 ng/mL)(25). Mazza reported ferritin levels between 276 and 8031 ng/mL(26). Berak reported ferritin levels 2171.5±1439.8 (103-5150 ng/m) (27). Mansouri-targhabeh reported ferritin levels 2597±1976 ng/mL (28).

Mean serum ferritin in patient with weight more than 5 percentile was 2309+/-1284, and with weight less than 5 percentile was 3199+/-1545 ( P-value=0.017). In patient with normal BMI, mean serum ferritin was 2679+/-1378, and it was 2596+/-1777 with low BMI. High serum ferritin levels during puberty cause delay of growth retardation and development in transfusion dependent thalassemia patients (29). 

The mean and S. D of ferritine in this research is similar to another research. Iron overload in patients with thalassemia is a common feature, which requires continuous chelation therapy and monitoring. Serum ferritin determination is widely accepted as a simple method for following iron load in patients with primary hemochromatosis. However, several reports on thalassemic patients emphasize that ferritinemia is not accurate. Direct measurement of iron in the liver (HIC) and magnetic resonance imaging (MRI) are more precise. 

A research on 33 thalassemic patients showed Ferritin levels ranged between 276 and 8031 ng/mL, and liver iron content ranged from 1.6 to 31.0 mg/g dry weight. Grade III or IV liver siderosis was recorded in 23/33 these patients, which they showed severe or very severe siderosis at MRI (24). Significant correlations with ferritin levels were recorded between grade IV and grades III, II and I (p < 0.01, p = 0.02, and p = 0.03, respectively).

The study shows that serum ferritin levels significantly correlated with the true status of hemochromatosis in thalassemic patients (24). In present study the mean age of starting blood transfusion was 9±6 months. Shamshirzan et al reported blood transfusion in 15.4months (19). Karimi reported 7±4 months. Studies in Germany reported 10 months (3-29 months) (30, 20). 

All of the patients with beta thalassemia major must start blood transfusion before 2 years old. The mean and S. D of deferoxamine dosage was 30 ± 11.16 mg / kg. One research in Hong Kong reported deferoxamine dosage between 20 -50 mg / kg (31). Yin reported iron chelator was used regularly in 44.6%, irregularly in 26.9%, and was not used in 28.5% of thalassemic patients. The most important consequence of life-saving transfusions in thalassemia is the inexorable accumulation of iron within tissues, causing progressive organ dysfunction. This is fatal without chelating therapy. Iron overload may be prevented or treated with a chelating agent capable of making complex with iron and promoting its excretion (18).

Forty six percent of our patients had hepatomegaly. In a study conducted by Berak etal showed that 50% of patient had hepatomegaly, 23.68% moderate splenomegaly, 25.79% sever splenomegaly (27). Severe hepatomegaly in thalassemic patients was seen in 90% of patients with normal thyroid hormone and 10% in patients with subclinical hypothyroidism in Kurdistan (32).

The association between iron overload and pathology of the liver in transfusion-dependent patients with β thalassemia major has been extensively studied. Viral hepatitis is an important cause of the acute and chronic hepatitis، cirrhosis and hepatocellular carcinoma. Because of the frequent transfusions، thalassemic patients are at a greater risk of developing these problems (33). 

The presence of liver fibrosis in patients with beta-thalassemia major has been demonstrated to be an important negative prognostic factor. Identification of liver fibrosis in early stage would have great value. Previous histopathology study showed liver fibrosis including stage I and stage II by biopsies in 80% of the patients (34). 

In present study, the mean and S. D of AST and ALT were 95 +/- 70 IU/L and 70 +/- 35U/L, respectively. Similar study by Company et al in Kurdistan on 40 patients with beta thalassemia major showed that mean SGOT levels in hypothyroid and normal patients were 38.7 ± 14.8 U/L and 50 ± 27.8 U/L (p= 0.2) respectively. So thyroid dysfunction could not be correlated with liver function or plasma ferritin level (32). 

A research on 104 patients with beta thalassemia major showed a significant correlation between iron level as indicated by transferrin saturation or serum ferritin levels and SGOT, SGPT levels. Abnormal liver function represented by elevated levels of SGOT, SGPT and serum alkaline phosphatase, which was observed more frequently in patients with iron overload than in patients with a lower level of iron (35). 

A study in Pakistan showed that 47% of their patients had an increased alkaline phosphatase, which might be attributable to liver disease (36). Shams et al showed that liver dysfunction in about 20% of the patients. AST and ALT levels were 37.2 ± 27.4 (8–168) and 24 ± 27.4 (6–165) (U/L) respectively (37). Another study in Pakistan on 48 diagnosed patients of β thalassemia major showed the mean of AST and ALT about 58 ± 6 and 64 ± 6 (U/L) respectively (38).

 A research on 99 patients with multiple transfusion in China showed the seropositive hepatitis C antibody patients had higher serum alanine aminotransferase, aspartate aminotransferase and ferritin concentrations (91 +/- 82 IU/L, 67 +/- 38 IU/L and 4797 +/- 2522 ng/ml respectively) than the seronegative patients (38 +/- 29 IU/L, 48 +/- 28 IU/L and 3620 +/- 2140 ng/ml respectively) (39). A study about blood borne viruses in Mashhad on 360 thalassemic patients showed that 30 (8.33%) had a positive anti-HCV antibody, and 8 patients (2.22%) had positive HBs antigen (28). 

## Conclusion

Hepatomegaly is one of the most findings in thalassemic patient that induced with hemosiderosis, extra medullary hematopoiesis, transmitted hepatitis B and C and cirrhosis. So starting of deferoxamine in the perfect time could prevent hemosiderosis**. S**erum ferritine and LFT were elevated in most patients in spite of defferoxamine pump use. It seems reevaluation of the current protocol of the defferoxamine administration needed.
